# Extracting Histologic Features to Distinguish Primary and Metastatic Squamous Cell Carcinoma of the Lung

**DOI:** 10.1111/pin.70084

**Published:** 2026-01-14

**Authors:** Yuri Tachibana, Andrey Bychkov, Kris Lami, Jijgee Munkhdelger, Hoa Pham, Thiyaphat Laohawetwanit, Zun Pwint Oo, Izumi Sato, Luka Brcic, Junya Fukuoka

**Affiliations:** ^1^ Department of Pathology Informatics, Graduate School of Biomedical Sciences Nagasaki University Nagasaki Japan; ^2^ Department of Pathology Kameda Medical Center Chiba Japan; ^3^ Department of Medical Laboratory Hospital of University of Medicine and Pharmacy Linh Dam Hanoi; ^4^ Chulabhorn International College of Medicine Thammasat University Pathumthani Thailand; ^5^ Department of Pathology University of Medicine Mandalay Myanmar; ^6^ Department of Clinical Epidemiology, Graduate School of Biomedical Sciences Nagasaki University Nagasaki Japan; ^7^ Department of Pathology Medical University of Vienna Vienna Austria; ^8^ Department of Pathology Hospital Graz II Graz Austria

**Keywords:** diagnostic formula, interstitial fibrosis, lung squamous cell carcinoma, primary versus metastasis, squamous dysplasia

## Abstract

Lung is a common site of metastasis for squamous cell carcinoma (SqCC), and distinguishing primary lung SqCC from pulmonary metastatic SqCC is critical for clinical decision‐making, including treatment planning. However, no practical histological criteria have been established for routine diagnosis. This study aimed to develop histopathological criteria to differentiate lung SqCC and pulmonary metastatic SqCC. A total of 85 surgical cases (48 primary and 37 metastatic) were collected with clinical background data. Seven histological features were evaluated. Six [morphological heterogeneity, dilated airway, interstitial fibrosis (IF), squamous dysplasia (SD), emphysema, RB macrophage] were presented as primary‐associated features, while stromal infarction was proposed as metastatic. Seven pathologists scored these features. Primary‐associated findings showed significantly higher score in primary cases. Tumor size (size) was significantly larger in the primary group (median: primary, 30 mm; metastatic, 14 mm; *p* < 0.001). A multivariate analysis incorporating size produced the “pathological primary formula” based on parameter estimates: 0.70・IF + 0.36・SD + 0.09・size, with an AUC of 0.86, sensitivity of 89.5%, and specificity of 70.2%. These results suggest that the extracted histologic features may provide reproducible criteria for distinguishing primary lung SqCC from pulmonary metastatic SqCC, offering insight into potential diagnostic applications.

## Graphical abstract

A scoring‐based formula was developed to distinguish primary from metastatic lung squamous cell carcinoma. Six features suggestive of primary and one of metastasis were scored from 0 to 2 by seven pathologists. The formula derived from multivariate analysis showed high AUC and sensitivity in both internal (*n* = 85) and external (*n* = 20) cohorts. Upper right: interstitial fibrosis; lower right: squamous dysplasia.

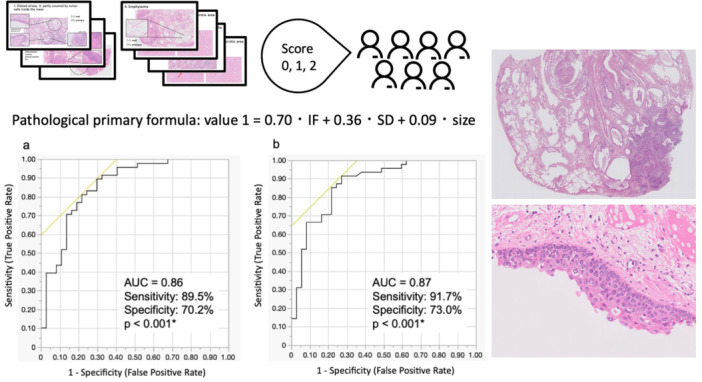

## 1 Background

Lung is a common metastatic site for squamous cell carcinoma (SqCC) originating from various organs throughout the body. Distant metastasis occurs in 1.5%–16.8% of head and neck SqCC cases, and among these, 53.1% involve the lung [1]. In Stage IV esophageal cancer, 77.3% of patients present with distant metastasis, with 9.7% of metastases occurring in the lung [2]. Furthermore, primary lung SqCC and SqCC in other organs share similar risk factors, such as smoking and alcohol consumption, which lead to a higher incidence of primary lung SqCC during follow‐up compared to patients without a prior SqCC history [3, 4, 5, 6].

Distinguishing whether lung SqCC is primary or metastatic is critically important for staging, prognosis, and treatment decision‐making. In cases where distant metastases are confirmed, surgical resection is typically avoided, and chemotherapy or radiation therapy is preferred [7]. On the other hand, if a lung lesion detected simultaneously is identified as the primary site, resection of both the primary and metastatic sites is recommended. Moreover, when the primary lesion is well‐controlled, resection of metastatic sites has been shown to improve prognosis [8]. Thus, determining whether lung lesions are primary or metastatic is an essential factor in therapeutic decision‐making. Nevertheless, despite various efforts to distinguish between primary and pulmonary metastatic lesions using imaging, immunohistochemistry, and genetic analyses, no histological approach with practical utility for routine diagnosis has been established to date.

According to previous reports, radiological approaches incorporating findings such as pleural indentation, ground‐glass opacity, central tumor localization, smoking history, and a history of malignancy have been shown to improve diagnostic accuracy [9, 10, 11]. However, in histological approaches, no definitive immunohistochemical (IHC) markers have been established to distinguish primary lung SqCC from metastatic lung SqCC [12]. While mutations in TP53 are frequently observed in SqCC, these mutations are not organ‐specific and are found in SqCCs from various sites, including the lung. P16 IHC, known as an HPV‐associated marker, shows nonspecific positivity in lung lesions, making it challenging to distinguish metastatic SqCCs from HPV‐associated SqCCs, such as those from oropharyngeal or cervical origins [13, 14].

In recent years, molecular marker‐based approaches have gained significant attention. Several studies have demonstrated that comprehensive genomic analyses can identify the primary site of origin with high accuracy in cancers of unknown primary origin, including SqCC [15, 16]. Jurmeister and colleagues developed a machine‐learning algorithm utilizing differences in DNA methylation patterns between primary lung SqCC and head and neck SqCC, achieving a diagnostic accuracy of 96.4% [17]. Additionally, software capable of identifying the primary organ of SqCC with a sensitivity of 91% has been introduced [18]. These methods hold great promise, particularly in diagnosing cancers of unknown primary origin, by assisting in determining treatment strategies. However, their utility in routine clinical practice is limited by financial constraints and turnaround time issues.

In this study, we aimed to explore whether histologic features could help distinguish between primary lung SqCC and pulmonary metastatic SqCC. This exploratory approach focuses on identifying reproducible morphological characteristics that may aid diagnosis in routine pathology practice.

## 2 Materials and Methods

### 2.1 Data Collection

Surgically resected lung specimens collected at a single institution were recruited, focusing on cases histologically diagnosed as SqCC. Cases diagnosed between 2003 and 2019 were included for the metastatic group. For primary cases, those diagnosed between 2018 and 2020 were selected. Metastatic cases were defined based on a comprehensive clinicopathological assessment. Specifically, the following criteria were required: (1) extrapulmonary primary SqCC confirmed both radiologically and histologically, and (2) tumor behavior and temporal relationship clinically consistent with metastatic spread. The seven histological features investigated in this study were not used in the case selection process. Additionally, for validation purposes, 20 cases were selected, comprising 15 primary SqCC cases and 5 metastatic cases.

### 2.2 Design

In this study, seven pathologists were asked to determine whether the tumor was primary or metastatic. The evaluation process consisted of two rounds: the first round used only hematoxylin and eosin (HE)‐stained slides, and the second round included seven specific histological findings. Additionally, the pathologists were asked to score these seven findings during the second round.

The seven participating pathologists included six general pathologists and one respiratory pathology specialist. For each case, one HE‐stained virtual slide was prepared, scanned using the PHILIPS Ultra Fast Scanner. The slides were selected to include areas with the widest tumor region, while ensuring the observation of tumor stroma and surrounding lung tissue.

In the first round, the pathologists were presented with HE‐stained slides for each case and asked to make a diagnosis of primary or metastatic tumor.

**Figure 1:**
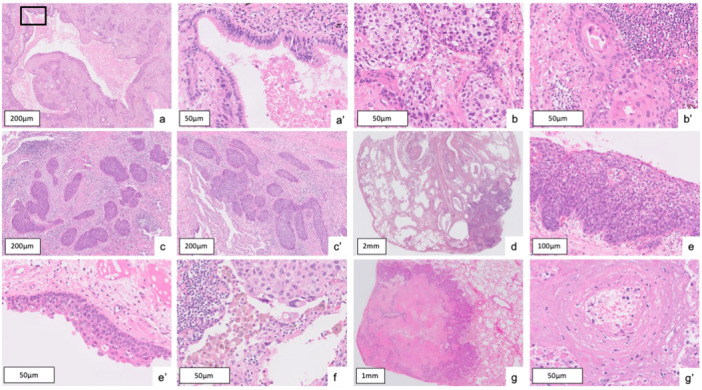
Features of histological findings for primary and metastatic cases. Primary findings: (a, a') dilated airway, morphological heterogeneity [(b) the primary case: some areas exhibited clear cells, while other areas (b') showed eosinophilic cytoplasm; (c, c') the metastatic case: all areas exhibited a similar morphology], (d) interstitial fibrosis and emphysema, (e, e') squamous dysplasia, and (f) RB macrophage. Metastatic finding: (g, g') infarction of the normal stroma.

In the second round, the seven histological findings related to primary or metastatic diagnosis were introduced (six findings associated with primary tumors and one associated with metastatic tumors). These histological findings were selected through expert (A. B., K. L., L. B., and J. F.) consensus discussions based on literature review and clinical relevance [19, 20, 21, 22]. The pathologists evaluated each of these findings for every case using a scoring system of 0, 1, or 2. A score of 0 was used when no discernible findings were observed. A score of 1 was applied when the findings were mild, incomplete, or limited in extent. A score of 2 was assigned when the findings were clearly present, extensive, and continuous. An example of a finding scored as 2 is shown in Figure 1.

The six primary tumor findings included: morphological heterogeneity (MH), dilated airway (DA) surrounding the tumor, interstitial fibrosis (IF) in nonneoplastic lung adjacent to carcinoma, squamous dysplasia (SD) of airway, emphysema (E), and respiratory bronchiole macrophage (RB) with pigment accumulation. The six primary tumor findings were defined as follows: morphological heterogeneity was characterized by structural and cytological diversity within the tumor, including variations in differentiation, basaloid morphology, or clear cell‐like change. Dilated airway referred to the dilation of bronchi or bronchioles adjacent to the tumor, while SD indicated atypical squamous proliferation of the airway epithelium. Respiratory bronchiole macrophage denoted the aggregation of pigmented macrophages within respiratory bronchioles, and IF and emphysema represented fibrotic thickening of the interstitium and enlargement of airspaces in the nonneoplastic lung, respectively. The single metastatic tumor finding was infarction of the normal stroma (IS), representing stromal necrosis caused by the tumor.

Representative images and explanations for these findings were provided to the pathologists in advance as part of training materials. After this preparation, the pathologists reassessed the cases with the additional information and made another diagnosis of primary or metastatic tumor for each case. To minimize subjective bias, the order of case presentation was shuffled between the first and second rounds, and new case numbers were assigned.

We also collected cross‐sectional data, including age, sex, smoking history, CT imaging findings (location and number of masses), and the primary organ in cases of metastasis.

### 2.3 Statistics

The pathological and background clinical findings of the cases were summarized using descriptive statistics. The relationships between clinical findings or pathological findings and the diagnosis of primary or metastatic tumors were evaluated using univariate analysis. For continuous variables, the Wilcoxon rank‐sum test was employed, and for categorical variables, Fisher's exact test was used. Statistical significance was defined as a two‐tailed *p* < 0.05.

Next, multivariate analysis was performed using ordinal logistic regression analysis with two analytical models: (1) Analysis using only pathological findings and (2) Analysis combining pathological findings and clinical findings. All variables used in the univariate analysis were included in the multivariate analysis.

Based on the results of these analyses, two formulas for calculating the Primary Score were developed for both models. Receiver operating characteristic (ROC) curves were drawn using the scores calculated by each formula, and the area under the curve (AUC) was determined to assess diagnostic performance. These analyses were conducted for both internal cases and validation cases. For the internal cohort, stratified 10‐fold cross‐validation was also performed to evaluate the robustness and generalizability of the model. To evaluate potential site‐specific bias, metastatic tumors were grouped according to their primary organ site. For each organ category, the proportion of cases predicted as metastasis by the formula was calculated and compared using Pearson's *χ* ^2^ test.

The agreement rate of diagnoses was evaluated using Fleiss' *κ* coefficient.

All statistical analyses were performed using JMP Clinical 17 (SAS Institute Inc., Cary, NC, USA).

## 3 Results

### 3.1 Patient Characteristics and Clinical Background

The subjects of this study consisted of a total of 85 cases, including 48 primary lesions and 37 metastatic lesions. The resection methods for the specimens included lobectomy (29 primary, 8 metastatic), segmentectomy (9 primary, 7 metastatic), wedge resection (9 primary, 20 metastatic), and other procedures (1 primary, 2 metastatic).

The clinical background of the cases is summarized in Table 1. The median age was 70 years (interquartile range [IQR]: 66–74) in the primary group and 68 years (IQR: 62–73) in the metastatic group, showing a significant difference (*p* = 0.037). Both groups had a higher proportion of males—40 cases (83%) in the primary group and 29 cases (78%) in the metastatic group (*p* = 0.587).

**Table 1:** Clinical findings.

			Primary	Metastasis	*p*
			*n* = 48, 56%	*n* = 37, 44%	
Age (median [IQR])			70 [66–74]	68 [62–73]	0.037*
			*n* (%)		
Sex	Male		40 (83%)	29 (78%)	0.587
Smoking status	Current/former		42 (88%)	24 (65%)	0.026*
	Never		6 (12%)	13 (35%)	
CT findings	Localization	Central	7 (15%)	1 (3%)	0.293
		Peripheral	40 (83%)	33 (89%)	
		Others	1 (2%)	3 (8%)	
	Number of mass	Solitary	44 (83%)	23 (62%)	0.001*
		Multifocal	4 (17%)	14 (38%)	
Primary site of metastasis cases	Pharynx		—	12 (32%)	
	Esophagus		—	8 (22%)	
	Uterine cervix		—	5 (14%)	
	Larynx		—	3 (8%)	
	Oral		—	3 (8%)	
	Skin		—	3 (8%)	
	Lung		48 (100%)	2 (5%)	
	Urinary bladder		—	1 (3%)	

*Note:* The breakdown and results of univariate analysis for primary and metastatic cases regarding clinical information are presented. Percentages are calculated based on the number of cases within each group (primary or metastatic SCC). Asterisks (*) indicate items that were determined to be statistically significant.

Regarding smoking history, most patients in both groups were smokers, with a higher tendency observed in the primary group (*p* = 0.026). The numbers of current or former smokers were 42 cases (88%) in the primary group and 24 cases (65%) in the metastatic group, while the numbers of never smokers were 6 cases (12%) in the primary group and 13 cases (35%) in the metastatic group.

For the location of lesions on CT, most lesions in both groups were located in the peripheral region, with no significant difference observed (*p* = 0.294). The distribution of lesions was as follows: central, peripheral, and others—primary group: 7 cases (15%), 40 cases (83%), and 1 case (2%), respectively; metastatic group: 1 case (3%), 33 cases (89%), and 3 cases (8%), respectively.

Regarding the number of lesions, the metastatic group showed a higher proportion of multifocal lesions (*p* = 0.001). Solitary lesions were observed in 44 cases (83%) in the primary group and 23 cases (62%) in the metastatic group, while multifocal lesions were observed in 4 cases (17%) in the primary group and 14 cases (38%) in the metastatic group.

The primary organs of the metastatic lesions were as follows: pharynx (12 cases, 32%), esophagus (8 cases, 22%), cervix (5 cases, 14%), larynx (3 cases, 8%), oral cavity (3 cases, 8%), skin (3 cases, 8%), intrapulmonary metastasis (2 cases, 5%), and bladder (1 case, 3%). All cervical cases in this study showed p16 positivity in both the primary and metastatic lesions.

### 3.2 Diagnostic Accuracy of Pathologists

The mean accuracy rate of the seven pathologists in diagnosing primary or metastatic tumors was 64.4% in the first round, where representative histological materials were not presented, and 65.4% in the second round, where the findings were presented, showing no significant difference. However, the Fleiss' *κ* value, which represents inter‐pathologist agreement, improved from 0.208 in the first round to 0.407 in the second round with the presentation of histological findings, indicating an improvement in diagnostic concordance.

### 3.3 Comparison of Histological Findings Between the Primary and Metastatic Group

Next, the results related to histological findings are summarized in Table 2. Tumor size tended to be larger in the primary group, with a significant difference observed (*p* < 0.001). Specifically, the median tumor size was 30 mm (95% CI: 27.3–38.0) in the primary group and 14 mm (95% CI: 13.3–20.6) in the metastatic group. In cases with multiple lesions, tumor size was defined as the maximum diameter.

**Table 2:** Histological findings.

		Primary		Metastasis		*p*
		Average	Median (IQR)	Average	Median (IQR)	
Tumor size (median [IQR], mm)		32.6	30 (20.5–40.0)	16.9	14 (9.0–21.0)	< 0.001*
Score of each histological finding (0, 1, 2)						
Primary findings	Morphological heterogeneity	0.71	1 (0–1)	0.50	0 (0–1)	< 0.001*
	Dilated airway	0.91	1 (0–2)	0.45	0 (0–1)	< 0.001*
	Interstitial fibrosis	0.79	1 (0–1)	0.36	0 (0–1)	< 0.001*
	Squamous dysplasia	0.90	1 (0–2)	0.45	0 (0–1)	< 0.001*
	Emphysema	0.69	1 (0–1)	0.56	0 (0–1)	0.021*
	RB macrophage	0.40	0 (0–1)	0.29	0 (0–1)	0.027*
Metastatic finding	Infarction of the normal stroma	0.28	0 (0–0)	0.32	0 (0–0)	0.503

*Note:* The breakdown and Wilcoxon rank‐sum test results for primary and metastatic cases are shown. The mean value for each finding was calculated as follows: the total score for each finding divided by the number of cases in the primary and metastatic groups, respectively. Asterisks (*) indicate items that were determined to be statistically significant.

**Figure 2:**
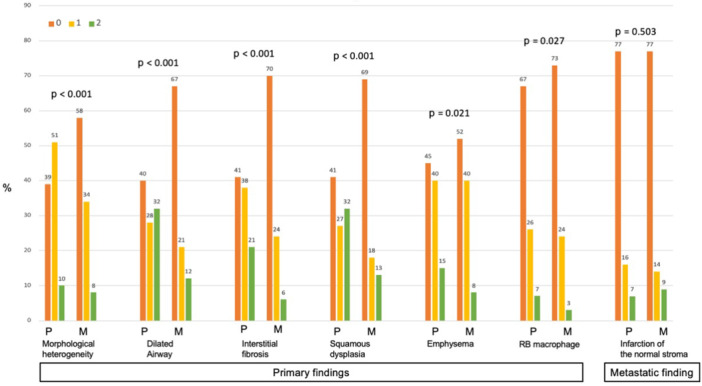
Distribution of scores assigned by seven pathologists for each finding. The distribution of scores (0, 1, 2) assigned for each finding is presented. The percentage (%) was calculated using the following formula: Percentage = number of cases with each score for each finding/85 cases・7 pathologists. P: primary cases, M: metastatic cases.

The primary histological findings we presented showed higher mean scores in the primary group, with significant differences observed. The mean scores and results of univariate analyses for the primary and metastatic groups were as follows: MH: 0.71 versus 0.5 (*p* < 0.001), DA: 0.91 versus 0.45 (*p* < 0.001), IF: 0.79 versus 0.36 (*p* < 0.001), SD: 0.90 versus 0.45 (*p* < 0.001), E: 0.69 versus 0.56 (*p *= 0.021), and RB: 0.40 versus 0.29 (*p* = 0.027). Furthermore, cases with high scores for primary histological findings were more frequently observed in the primary group (Figure 2).

In contrast, there were no significant differences between the two groups in metastatic findings, with mean scores of 0.28 versus 0.32 (*p* = 0.503).

### 3.4 Multivariate Analysis of Pathological and Clinical Findings

The results of the multivariate analyses using pathological findings alone and a combination of pathological and clinical findings are summarized in Table 3.

**Table 3:** Multivariate analysis.

	Pathology‐only analysis		Combined pathology and clinical analysis	
	Parameter estimate	*p*	Parameter estimate	*p*
Tumor size	−0.09	< 0.001*	−0.10	< 0.001*
Morphological heterogeneity	−0.03	0.866	−0.16	0.415
Dilated airway	−0.27	0.080	−0.27	0.139
Interstitial fibrosis	−0.70	< 0.001*	−0.65	< 0.001*
Squamous dysplasia	−0.36	0.012*	−0.15	0.355
Emphysema	−0.17	0.322	−0.22	0.259
RB macrophage	0.02	0.909	0.07	0.762
Stromal component	0.07	0.714	0.09	0.668
Age			−0.07	< 0.001*
Sex			0.48	0.010*
Smoking status			0.19	0.268
Location			−0.55	0.057
Number of mass			0.59	0.001*

*Note:* Multivariate analysis was performed using pathological findings alone and in combination with clinical findings. The parameter estimates derived from this analysis were used to create the primary score formula. Asterisks (*) indicate items that were determined to be statistically significant.

In the analysis using pathological findings alone, significant differences were observed for tumor size (*p* < 0.001), IF (*p* < 0.001), and SD (*p* = 0.012).

In contrast, the analysis combining pathological and clinical findings revealed significant differences for tumor size (*p* < 0.001), IF (*p* < 0.001), age (*p* < 0.001), sex (*p* = 0.010), and number of masses (*p* = 0.001).

### 3.5 Development of Predictive Models for Primary Tumors

Based on these results, two formulas were developed to calculate primary scores by assigning approximated coefficients to the scores of the parameters with significant differences:

1. Pathological primary formula (Formula 1):
  value 1 = 0.70・IF + 0.36・SD + 0.09・size
2. Pathological and clinical primary formula (Formula 2):
  value 2 = 0.65・IF + 0.10・size + 0.07・age − 0.48・sex − 0.59・number of mass.

### 3.6 Analysis of Training Cohort Cases

**Figure 3:**
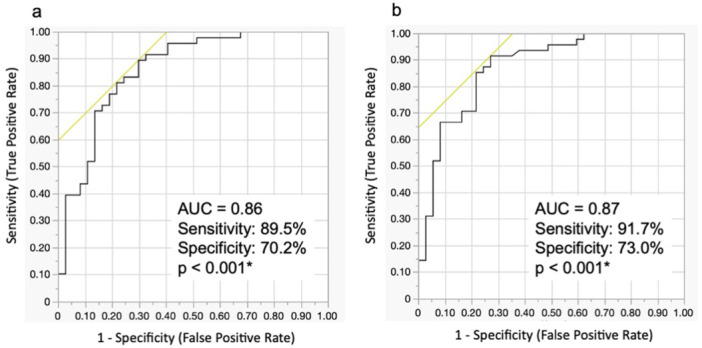
ROC curves showing correlation between primary score formulas and primary/metastatic SqCC. (a) Created using pathological findings only. (b) Created using both pathological and clinical findings. Each curve shows sensitivity and specificity for cutoff values of 2.13 and 6.71, respectively.

Using Formula 1, the analysis of 85 training cohort cases yielded a AUC of 0.86. With a cutoff value of 2.13, the sensitivity was 89.5% and the specificity was 70.2% (*p* < 0.001). In contrast, using Formula 2, the AUC was 0.87. With a cutoff value of 6.71, the sensitivity was 91.7% and the specificity was 73.0% (*p* < 0.001). The ROC curves for these analyses are shown in Figure 3. To evaluate the robustness and generalizability of the models, stratified ten‐fold cross‐validation was performed, treating primary tumors as the positive class. Formula 1 achieved a mean AUC of 0.86 with a sensitivity of 84.0% and specificity of 70.0, while the pathology + clinical model yielded a mean AUC of 0.81 with a sensitivity of 92.0% and specificity of 45.8%. These findings indicate that the predictive performance remained stable across different data partitioning strategies. In addition, we assessed whether tumors from specific primary sites were more likely to be misclassified by the formulas. When metastatic cases were stratified by the primary tumor site, the proportion of cases classified as metastasis was as follows: skin (100%, 3/3), tongue (100%, 2/2), urinary bladder (100%, 1/1), pharynx (75.0%, 9/12), esophagus (62.5%, 5/8), uterine cervix (60.0%, 3/5), larynx (33.3%, 1/3), lung (0%, 0/2), and oral cavity (0%, 0/1). However, there was no significant difference in misclassification tendency among primary sites (Pearson's *χ* ^2^ = 8.320, *p* = 0.4029), indicating that the formula did not exhibit a site‐specific bias.

### 3.7 Analysis of Validation Cases

Subsequent analysis of 20 validation cases showed that using Formula 1 resulted in an AUC of 0.83. With a cutoff value of 3.65, the sensitivity was 60% and the specificity was 100% (*p* = 0.020). For Formula 2, the AUC was 0.89. With a cutoff value of 7.82, the sensitivity was 80% and the specificity was 100% (*p* = 0.020).

## 4 Discussion

In this study, we aimed to develop a diagnostic support tool for distinguishing between primary lung SqCC and pulmonary metastatic SqCC by analyzing the impact of combining pathological findings and clinical information on diagnostic performance. Analyses were conducted on 85 training cohort cases and 20 validation cases, yielding several key insights.

We identified reproducible histologic features that can assist in distinguishing between primary and metastatic pulmonary SqCC, rather than proposing a definitive predictive model. The primary score formula derived using multivariate analysis—Formula 1, based on pathological findings alone, and Formula 2, which combines pathological and clinical findings—both demonstrated high diagnostic performance. The AUC for internal cases was 0.86 with Formula 1 and 0.87 with Formula 2, indicating strong diagnostic accuracy. Notably, Formula 2 showed improved sensitivity (91.7%) and specificity (73.0%) by incorporating clinical information, suggesting that the inclusion of clinical data contributes to enhanced diagnostic precision. In the validation cases, Formula 2 outperformed Formula 1 in diagnostic performance, with AUCs of 0.89 and 0.83, respectively, highlighting its potential utility in real‐world clinical applications.

Regarding each histological finding, all primary findings (MH, DA, IF, SD, E, RB) showed significantly higher scores in the primary group, confirming their ability to accurately characterize primary lesions. On the other hand, the metastatic finding (IS) did not show a significant difference between the two groups but tended to be slightly higher in the metastatic group. These findings indicate that the histological markers are useful for distinguishing between primary and metastatic lesions.

MH reflects the nature of the tumor itself. Primary tumors exhibit greater morphological diversity, whereas metastatic tumors tend to consist of homogenous tumor cells that have selectively proliferated to adapt to the environment of the metastatic site [23]. This characteristic, which is specific to primary SqCC, was supported by the results of this study. Regarding DA as a finding around the tumor, it is known that primary SqCC grows predominantly around the airway epithelium in a replacement pattern [24]. During tumor growth, the existing bronchi are pulled, resulting in airway dilation, which is more prominent in primary SqCC. This observation aligns with the findings of this study. SD, a well‐known precancerous condition of SqCC, was also significantly observed in the primary group in this analysis [25]. E and RB are findings associated with smoking, and the inclusion of smoking history in the analysis of this study reinforces this association [26, 27]. Furthermore, it is well‐established that IF in the context of combined pulmonary fibrosis and emphysema (CPFE) is associated with an increased risk of SqCC [19]. The results of this study are consistent with this knowledge. Previous studies have shown that peripheral SqCC in the lung often develops against a background of fibrosis, and the present results also highlight histological findings specific to SqCC [19]. Notably, infarction of the normal stroma (IS) is observed in cases of hematogenous metastasis, where tumor emboli can cause infarction [28]. This leads to coagulative necrosis associated with IS, which is considered a finding suggestive of metastatic lesions.

Regarding diagnostic concordance, the mean accuracy rate in training cohort cases showed no significant change before and after the presentation of histological findings (64.4% vs. 65.4%). However, the Fleiss' *κ* coefficient improved from 0.208 to 0.407, indicating that the presentation of histological findings enhanced diagnostic consistency among pathologists. This result underscores the importance of histological information in the diagnostic process.

In the multivariate analysis focusing on pathological findings, only IF, SD, and size remained as major diagnostic factors. When clinical findings were included in the analysis, only IF and size were retained as significant factors. These results suggest that interactions between the selected factors and other excluded factors may have influenced the outcomes. Notably, the relationship between smoking‐associated IF and emphysema is known as CPFE. Additionally, RB macrophage and squamous metaplasia are well‐recognized findings associated with smoking [20]. Furthermore, SD is known to develop on a foundation of squamous metaplasia, suggesting that these findings may be interconnected through a series of mechanisms [21]. Additionally, DA is also known as a finding associated with fibrosis in interstitial pneumonia [22]. Primary pulmonary SqCC may develop on a background of fibrotic tissue and expand by pulling and dilating the surrounding bronchi during its growth process. As a result of these influencing factors, IF, SD, and size were identified as major diagnostic factors in the multivariate analysis. These findings suggest that fibrosis and bronchial epithelial changes may play an important role in the development and diagnosis of primary SqCC.

The results of this study suggest that integrating pathological and clinical findings may improve the diagnostic accuracy for distinguishing primary SqCC from metastatic SqCC. However, this study has several limitations. First, the sample size was relatively small, and all cases were collected from a single institution, which limits the generalizability of the findings. Future studies should involve a larger number of cases from multiple institutions to validate these results. Second, the definition of metastatic cases in this study was based on clinicopathological evaluation, and molecular‐level analyses, such as genetic studies, were not included. A more comprehensive approach incorporating molecular analysis is necessary in future research to ensure accurate diagnosis of metastatic cases. Third, while this study focused primarily on pathological findings, no integrated analysis with imaging findings was conducted. Incorporating imaging data could potentially further enhance diagnostic accuracy. Given these limitations, future research should adopt a multifaceted approach to develop more accurate and widely applicable diagnostic tools.

The histological findings evaluated in this study are based on simple observations of HE‐stained specimens, requiring no special staining, immunostaining, or costly genomic analysis. This simplicity allows for immediate application in routine clinical settings without specialized equipment. Based on the primary formula derived in this study, further development of web‐based applications is expected to facilitate clinical implementation, as demonstrated in our previous work on a transbronchial lung biopsy model for classifying benign and malignant lesions (Figure S1) [29]. These findings may support efficient differentiation between primary and metastatic SqCC, contributing to improved diagnostic accuracy in pathology.

## 5 Conclusion

We successfully extracted morphological features that can help distinguish between primary and metastatic SqCC by observing H&E slides.

## Author Contributions

Conception and design of the study: Yuri Tachibana and Junya Fukuoka. Acquisition of data: Yuri Tachibana. Evaluation of histological features: Kris Lami, Jijgee Munkhdelger, Hoa Pham, Thiyaphat Laohawetwanit, Zun Pwint Oo, and Luka Brcic. Analysis of data: Yuri Tachibana and Izumi Sato. Drafting the manuscript or figures: Yuri Tachibana. Manuscript review and editing: Kris Lami, Andrey Bychkov, and Junya Fukuoka.

## Conflicts of Interest

The authors declare no conflicts of interest.

## Supporting information


**Supporting Figure 1:** Example of Clinical Application: Output of Artificial Intelligence (AI)‐Based Classification for Transbronchial Lung Biopsy (TBLB).

## Data Availability

The data that support the findings of this study are available on request from the corresponding author. The data are not publicly available due to privacy or ethical restrictions.
