# Supplementary figures and images for: Extracting Histologic Features to Distinguish Primary and Metastatic Squamous Cell Carcinoma of the Lung
Source: Pathol Int. 2026 Jan 14;76(1):e70084. doi: 10.1111/pin.70084 (PMC12835960; doi:10.1111/pin.70084)

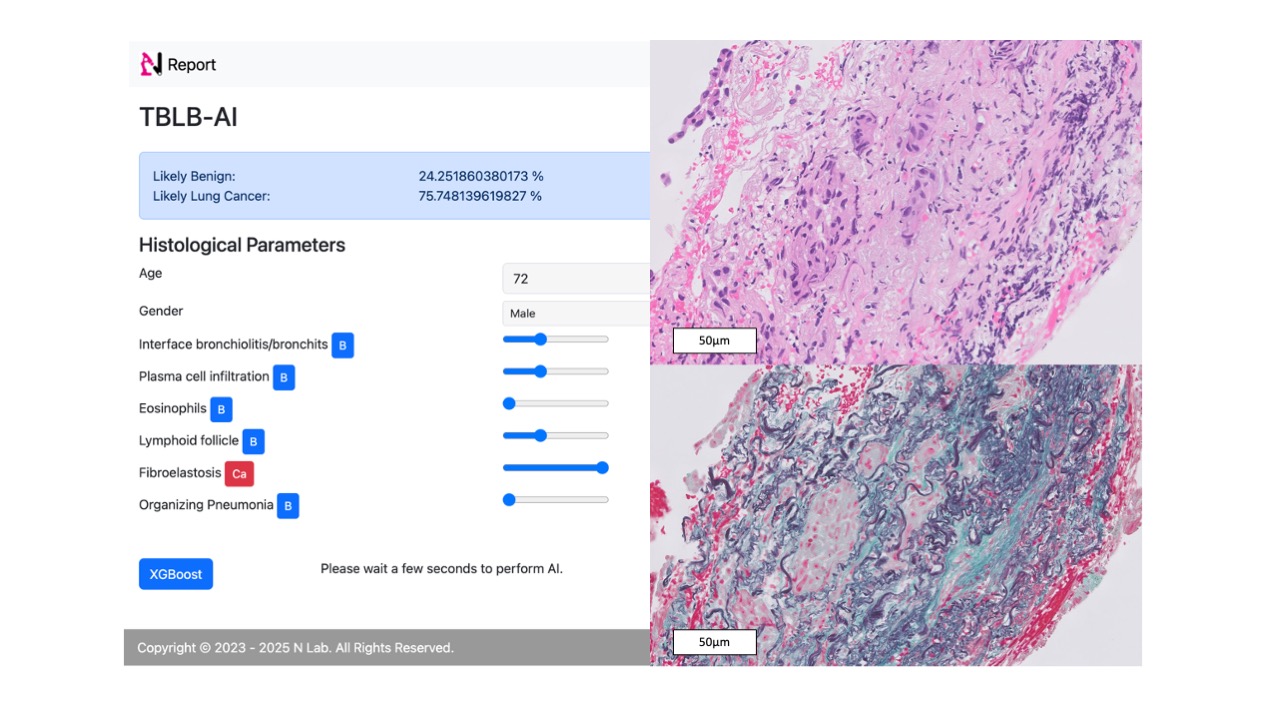

Supplement: Supplementary file 1 — Supporting Figure 1: Example of Clinical Application: Output of Artificial Intelligence (AI)‐Based Classification for Transbronchial Lung Biopsy (TBLB). [file PIN-76-0-s001.jpg]
